# Discrimination and Calibration Properties of the Violence Risk Appraisal Guide–Revised in a Not Criminally Responsible Provincial Population

**DOI:** 10.1177/10731911221116564

**Published:** 2022-08-29

**Authors:** Robi L. Wirove, Mark E. Olver, Andrew Haag

**Affiliations:** 1 The Ottawa Hospital, ON, Canada; 2 University of Saskatchewan, Canada; 3 Alberta Hospital Edmonton, Canada; 4 University of Alberta, Edmonton, Canada

**Keywords:** Violence Risk Appraisal Guide–Revised, violence risk assessment, actuarial, recidivism, not criminally responsible on account of mental disorder

## Abstract

This study examined the discrimination and calibration properties of the Violence Risk Appraisal Guide–Revised (VRAG-R) within a large subset of the population of 574 individuals who had been found Not Criminally Responsible on Account of Mental Disorder (NCRMD) in Alberta. The VRAG-R was scored on all individuals identified via *The Alberta NCR Project* database from every file that contained sufficient relevant information and recidivism data were obtained via official criminal records. The VRAG-R demonstrated strong discrimination properties for general and violent recidivism over 5-year, 10-year, and global follow-ups. Calibration analyses, however, indicated that the VRAG-R substantially over estimated violence risk and that there was poor agreement between expected and observed recidivism rates for this population. When examined in the male subsample, these issues remained but to a lesser degree; examination of VRAG-R discrimination and calibration for females was not possible due to a lack of recidivists. Results indicated strong discrimination but poor calibration properties of the VRAG-R in this NCRMD population. Overall, the results support the use of the VRAG-R within a population of persons found NCRMD when employed in tandem with other measures as part of a comprehensive psychological risk assessment.

Persons with mental health diagnoses can come into conflict with the law for several reasons, one of which is by way of the commission of criminal acts that are attributable to active mental health symptoms. Although the commission of severe violence is infrequent among persons with mental disorder (Douglas et al., 2009), the international news media is nonetheless replete with high profile cases of tragic and extremely violent acts committed by mentally ill persons (Stuart, 2006; Wahl, 1992). In some instances, these persons will come under forensic mental health jurisdiction, such as through insanity defense legislation, and a series of detention, discharge, and risk management decisions will by necessity be made by review boards to promote public safety and client wellbeing. Part of this vital task is the ability to accurately assess a person’s risk for recidivism and particularly future violence, to inform discharge decisions and postrelease risk management.

Increasingly, the use of structured risk assessment measures has become a mainstay in forensic mental health. The imposition of structure, whether the tool be actuarial and numeric (i.e., with scored linked to recidivism estimates) or structured and non-numeric (i.e., as with structured professional judgment or SPJ), structured risk assessment measures help to minimize human judgment biases, increase the fairness and accuracy of decisions, and increase clinical utility, for instance, by informing service intensity and treatment foci for risk management (Brown & Singh, 2014). This study examined the predictive properties of a structured violence risk assessment measure developed on, and frequently used with, forensic mental health samples—the Violence Risk Appraisal Guide–Revised (VRAG-R; Rice et al., 2013)—a population of men and women from the province of Alberta who received an insanity defense (i.e., Not Criminally Responsible on Account of Mental Disorder [NCMRD]) verdict over a 70-year catchment period and followed-up in the community post discharge. We turn to a brief review of the Canadian insanity defense legislation and the discharge and recidivism patterns of persons with a positive verdict, followed by a review of violence risk assessment considerations with this population and the VRAG-R measure.

## The Insanity Defense in Canada: Background and Context

The Canadian insanity defense is referred to as NCRMD in Section 16 of the *Canadian Criminal Code* (CCC). Persons can be declared NCRMD if they are determined by the courts to have been suffering from a disease of the mind (i.e., mental disorder broadly defined) at the time of the crime that (a) impaired their capacity to appreciate the nature or quality of a criminal act/omission or (b) that impaired the ability of the accused to know that the criminal act/omission was wrong. Most persons with mental illness who commit crimes are criminally processed through the traditional channels in the legal system, with the incident rate of NCRMD verdicts (2005–2012) approximately 7.5–9.1 per 10,000, representing about 0.1% of criminal court cases on an annual basis (Statistics Canada, 2014). The finding of NCRMD is unique as it represents neither a guilty plea nor a finding of innocence (Latimer & Lawrence, 2006). Instead, it represents a third option, wherein pursuant to CCC Section 672.38, an accused who is found to be NCRMD is diverted to a provincial or territorial review board unless they are immediately granted an absolute discharge by the court (Criminal Code, R.S.C. 1985, c. C-46, s.16). Once under the jurisdiction of a review board, an accused found NCRMD may be granted one of three dispositions outlined in CCC Section 672.54: (a) absolute discharge, (b) conditional discharge, or (c) detention in custody of a hospital. In the case of an absolute discharge, the accused is no longer under review board jurisdiction and returns to the community with no lingering restrictions on his or her liberty. Second, when granted a conditional discharge, an accused may be supervised in the community with restrictions placed upon their liberty under review board jurisdiction as deemed appropriate. Third, under detention, the accused is detained within the confines of a hospital for treatment and stabilization unless granted some form of release by the review board (Criminal Code, R.S.C. 1985, c. C-46, s.16; Latimer & Lawrence, 2006).

Review boards have the unwieldy task of generating a disposition that most balances the person’s right to freedom and the safety of the public. The discretion with which review boards can serve this function, however, changed with the imposition of new legislation instructing review boards to place public safety as the paramount consideration in review board hearings (Bill C-14, 2014). In response to increasing public pressure to ensure public safety and mental health, in 2014, Bill C-14, *The Not Criminally Responsible Reform Act* officially came into effect and made several amendments to the existing legislation, the most significant being the creation of a “high-risk accused” designation (Bill C-14, 2014). There is skepticism over whether there is evidence to support the creation of the high-risk accused designation (Goossens et al., 2019; Grantham, 2014; Haag et al., 2021; Lacroix et al., 2017). For instance, Goossens et al. (2019) found the high-risk accused designation to be more closely linked to index offense severity as opposed to public safety risk. Moreover, Charette et al. (2015) found that persons declared NCRMD for severe index offenses actually had lower rates of recidivism than persons who had committed less severe violent offenses or even nonviolent crimes. These considerations all underscore the necessity of structured violence risk assessment with persons found NCRMD.

## Violence Risk Assessment with Persons Found NCRMD

NCRMD cases frequently involve crimes against the person. According to Statistics Canada (2014), this represented about two-thirds (63%) of NCRMD cases between 2005 and 2012; 20% of which represented major assaults. Crocker et al.’s (2015) National Trajectory Project (NTP) of discharge and recidivism patterns of NCRMD cases from British Columbia, Ontario, and Quebec found similar results with 64.9% of the index offenses involving crimes against the person. Livingston et al. (2003) examined an NCRMD cohort within British Columbia and found that assault was most serious offense for about half (45.5%) of the cohort, while in Alberta, Haag et al. (2016) found that 46.9% of NCRMD index offenses were for nonsexual violent offenses (excluding homicide), 18.5% for homicide, and 10.6%, attempted homicide. Furthermore, recidivism rates of NCRMD patients are lower than that typically found in correctional samples in Canada and internationally, which would be consistent with NCRMD samples generally being lower risk and also the possibility of good discharge decisions and risk management activities by review boards (Fazel et al., 2016; Friendship et al., 1999; Goossens et al., 2019; Grann et al., 2008; Hayes et al., 2014; Norko et al., 2016; Richer et al, 2018; Simpson et al., 2018; Tabita et al., 2012). For instance, Charette et al. (2015) found the 3-year recidivism rates of discharged NCRMD cases to be 17% following an absolute discharge immediately postindex offense, 22% following a conditional discharge, and 22% following absolute discharge after a period under review board jurisdiction. In all, although having a violent index offense is a consistent theme within this population, index offense severity is not to be taken as evidence that a person found NCRMD will continue to pose a danger to public safety (*Winko v. British Columbia*; Forensic Psychiatric Institute, 1999). Such a determination should be made through formalized violence risk assessment.

The selection of one or more risk instruments to appraise violence risk involves as much the purpose and context of the assessment as it does the psychometric properties and intended function of any assessment measured used. Ultimately the purpose of violence risk assessment is violence prevention; this is achieved when individuals at high probability for future violence can be accurately identified and then given the greatest priority for risk management interventions to prevent future violence. The ability for a risk instrument to be able to aid such a preventive function, however, requires that it can predict the outcome of interest with satisfactory accuracy. To this end, the primary predictive properties of risk tools are discrimination and calibration (Helmus & Babchishin, 2017). Discrimination (relative risk) considers how risky an individual case is relative to other cases on a risk measure and can be examined through risk ratios, percentile ranks, or receiver operator characteristic (ROC) area under the curve (AUC) analyses. Such metrics enable examination of the extent to which risk scores on a given measure can accurately discern would-be recidivists from nonrecidivists and hence, higher risk from lower risk cases.

Calibration (absolute risk) “is a specific component of accuracy that measures how well a probabilistic prediction of an event matches the true underlying probability of the event” (Lindhiem et al., 2020, p. 840). An example of calibration is the use of logistic regression to generate rates of recidivism associated with individual scores (and hence groups of scores) on a risk measure. Furthermore, the Hosmer–Lemeshow goodness of fit test, can be conducted in logistic regression as a measure of calibration to examine to what extent the observed event (i.e., recidivism) match the expected event rates, with close correspondence between the two and a nonsignificant test indicating good calibration for the predictive model (Lindhiem et al., 2020). A further illustration of calibration would be to what the extent the expected recidivism rates generated from the risk categories in a reference group correspond to those observed in a comparison group, known as the *E*/*O* index (Hanson, 2017). These metrics are helpful as they represent the rates of recidivism associated with risk scores to inform decision making (e.g., conditional release, preventive detention), as well as to what extent the recidivism rates associated with risk scores from the normative sample generalize to other samples or settings elsewhere where the tool may be used.

Several risk instruments, both general and crime specific (e.g., sexual, violence, intimate partner violence), containing static and/or dynamic risk items, have been developed with evidence demonstrated for their predictive properties from meta-analysis (Campbell et al., 2009; Hanson & Morton-Bourgon, 2009; Yang et al., 2010). The VRAG (Harris et al., 1993) and Sex Offender Risk Appraisal Guide (SORAG; Quinsey et al., 1998) refer to a pair of empirical actuarial tools designed to evaluate risk for future violence among violent offending and sexual offending-specific populations. The measures were each developed and validated on a hybrid sample of largely NCRMD (or equivalent previous legislation) cases and individuals on remand awaiting trial and sentencing (Harris et al., 2015). The measures comprised static risk variables (e.g., offense history, demographic, and clinical), differentially weighted based on the magnitude of the predictor criterion associations of the best-predicting linear combination of variables.

Since its development more than two decades ago, the VRAG has been independently validated more than 60 times in correctional and forensic mental health samples in several countries (Rice et al., 2013). Results from meta-analytic reviews demonstrate moderate to large effects (AUC equivalents = .66–.73) for the VRAG’s predictive accuracy for future violence (Campbell et al., 2009; Hanson & Morton-Bourgon, 2009; Yang et al., 2010). The VRAG is used regularly in the United States and in other countries around the globe (Cox et al., 2018; Singh et al., 2014), and in Canada, it is often used in risk assessments with persons found NCRMD to assist with review board decisions (Wilson et al., 2015).

## The VRAG-R

The VRAG-R (Rice et al., 2013) was developed by integrating the VRAG and SORAG into one common violence risk assessment measure and then refining the item content. It has 12 items as with the VRAG, but is easier to score, for instance, using the antisocial facet from the Psychopathy Checklist-Revised (PCL-R; Hare, 1991, 2003) in place of the full scale and thereby at least partially addressing the “scale within a scale” criticism; however, critics may note that a prominent component of the PCL-R is still included and that the “scale within a scale” issue still applies, at least to some degree. The measure has also removed counterintuitive and controversial variables (e.g., inverse weighting of index offense homicides or schizophrenia diagnoses). Items are summed to generate total scores that are organized into nine risk bins; the risk bins are arranged into deciles with relatively equal proportions of cases within.

Psychometric research following on Rice et al.’s (2013) construction and validation of the measure has supported the predictive properties of the VRAG-R for future community violence in general correctional samples (AUC = .66, Glover et al., 2017), corrections-based sexual offending samples (AUC = .75, Gregório Hertz et al., 2021; AUC = .73, Olver & Sewall, 2018), and a forensic mental health sample (AUC = .74, Hogan & Olver, 2019). Cross-validation research on the VRAG-R’s calibration properties is needed. For instance, the observed 5-year rate of violent recidivism in the top bin (bin 9) of the VRAG-R was 80%, and the 15-year rate, 91%. Although the bins are now more representative in size and grouping, these are exceptionally high violent recidivism rates, and it is unclear to what extent such rates could generalize to other jurisdictions or samples. For instance, Olver and Sewall (2018), in a broadly high risk-need sexual offending sample, had a 5-year rate of violent recidivism of 53.9% for bin 9; this disparity (*E*/*O* index = 1.49 or *O*/*E* index = .67) represented significant overprediction by the VRAG-R norms. Moreover, 5-year rates of violence were nonsignificantly overpredicted for each of the remaining bins, despite the Olver and Sewall (2018) sample being significantly higher risk by approximately two-thirds of a standard deviation compared with the normative sample, (VRAG-R *M* = 10.2 vs. 3.6, respectively).

Most VRAG and VRAG-R research has been conducted with male forensic and correctional populations, and to our knowledge, only the original VRAG has been formally examined with female populations. In two prison-based samples, one from the United Kingdom (Coid et al., 2009) and one from Germany (Eisenbarth et al., 2012), VRAG scores had moderate-to-high predictive accuracy for general recidivism (AUCs = .66 and.72, respectively), and moderate accuracy for future violence (AUC =.65; Coid et al., 2009). Moreover, in a U.S. jail sample of 145 female inmates (Hastings et al., 2011), the VRAG was a poor predictor of institutional misconduct but had small-to-moderate predictive accuracy (AUCs = .61–.66) for 1-year rearrest postrelease. Two of the aforementioned studies (Coid et al., 2009; Hasting et al., 2011) also found women to have significantly lower VRAG scores than men.

## Current Study

Review boards make frequent use of violence risk assessment to inform discharge and management decisions within forensic mental health systems, and historically, the VRAG has been used frequently and evaluated heavily in settings around the world. The VRAG-R has a number of important advancements beyond the VRAG (and SORAG), however, further cross-validation research on the measure’s discrimination and calibration findings is needed, particularly forensic mental health samples, such as those with a positive insanity verdict. To date, there has yet to be a large-scale examination of these predictive properties of VRAG-R scores in an exclusive NCRMD population. Given that NCRMD cases tend to be lower risk with lower rates of recidivism, yet the VRAG measures are frequently used with this population, the issue of calibration and the generalizability of VRAG-R norms is paramount.

As such, this study examined the discrimination and calibration properties within the Alberta NCRMD population. There were four primary sets of hypotheses:

1. Females found NCRMD will have lower VRAG-R risk scores and bin number frequency distributions than their male counterparts.
2. VRAG-R bin and total scores will demonstrate moderate to high predictive accuracy (AUCs ≥ .64–.71), and hence strong discrimination properties for general and violent recidivism.
3. With respect to calibration, VRAG-R 5-year recidivism rates, as a function of VRAG-R bin and total score, will be higher for the Rice et al. (2013) normative sample than the Alberta NCRMD population.
4. The discrimination properties of the VRAG-R will extend to broad diagnostic groups (e.g., psychosis, antisocial personality disorder). In terms of calibration, higher rates of 5- and 10-year violent recidivism will be observed as a function of increasing VRAG-R score across diagnostic groups.

## Method

### The Alberta NCR Project

Ethics approval for this study was obtained through two university behavioral research ethics boards in neighboring provinces and operational approval was obtained from the Alberta Health Authority. Per Simmons et al. (2012), “We report how we determined our sample size, all data exclusions (if any), all manipulations, and all measures in the study” (par. 6). *The Alberta NCR Project* provided the foundation for this study. The Alberta NCR Project is an archivally based program of research with information sources that include Alberta Review Board dispositions, a provincial hospital internal database of patient demographic information, official reports from collateral sources, and psychological and psychiatric reports from community outpatient services. This program of research included every person declared NCRMD who has come under the Alberta Review Board’s jurisdiction since 1941. The program of research and its database is continually updated for new entries and recidivism data when available. Variables coded include sociodemographic information (e.g., age, sex, race, education level, marital status), clinical information (e.g., psychiatric diagnosis at time of disposition) and criminological information (e.g., date of NCRMD verdict, location of offense(s), index offense information, yearly warrant status, conditional discharge status, recidivism), and violence risk assessment measures, including the VRAG-R.

### Participants

As of October 2018, there were 574 identified cases of persons found NCRMD (male 83.1%, *n* = 477; female 16.9% *n* = 97). The participants were largely White (72.3%, 410/567; Indigenous 9.2%, 52/567; Other 18.5%, 105/567), early middle aged (*M* = 35.2 years, *SD* = 12.6, range age 15–83), single (83.2%, 472/567), and had not completed high school (68.0%, 372/547). Most of the sample had been diagnosed with a psychotic illness (84.5%, 453/539), followed by substance use disorder or SUD (56.2%, 257/457), mood disorder (38.7%, 179/462), and antisocial personality disorder or ASPD (24.5%, 107/437), which were the most common diagnoses. Most participants in the sample (60.8% 256/421) received more than one of these four diagnoses. The most frequent comorbidity pairings were with an SUD (i.e., dual diagnosis), which characterized 55.0% (204/371) of persons with psychosis diagnoses, 51.4% (73/142) of mood disorder diagnoses, and 77.8% (77/99) of ASPD diagnoses, which had this information available. Sufficient file information was available to score the VRAG-R on *N* = 478 cases (see Procedure for details).

### VRAG-R

The VRAG-R (Rice et al., 2013) is an empirical actuarial violence risk assessment tool statistically developed from a violent mentally disordered offender population in Ontario, Canada; approximately half the men were found NCRMD while the remainder were on remand for violent crimes and undergoing assessment (Harris et al., 2015). A more streamlined and user-friendly revision of its predecessor, the VRAG-R comprises 12 static items: (a) Lived with parents until age 16 (weight range −2 to +2), (b) Elementary school maladjustment (weight range −3 to +4), (c) History of alcohol or drug problems (weight range −2 to +4), (d) Marital status at time of index offense (weight range −1 to +1), (e) Criminal nonviolent history score (weight range −3 to +5), (f) Failure on conditional release (weight range −2 to +4), (g) Age at index offense (weight range −7 to +2), (h) Criminal violent history score (weight range −2 to +4), (i) Prior admissions to correctional institutions (weight range −2 to +6), (j) Conduct disorder prior to age 15 (weight range −2 to +5), (k) Sex offending history (weight range −2 to +3), and (l) Antisociality—facet 4 of the PCL-R (weight range −6 to +6). Individual items are weighted based on the strength and direction of their associations with violent recidivism. Items are summed to generate a total score ranging from −34 to +46 and arranged into nine risk bins. The measure is scored by a service provider for a given correctional/forensic client and can be completed solely from sufficiently detailed archival information sources (e.g., psychological assessments, criminal records, police reports, institutional behavioral records, social history intake), using the detailed scoring rules for the instrument (Harris et al., 2015) or as provided from www.vrag-r.org (n.d.).

### Recidivism Criteria

Recidivism was defined any as new criminal code conviction post discharge and was coded from Fingerprint Service (FPS) sheets through the Canadian Police Information Center (CPIC) as of 2015. Two operationalizations of recidivism were used. Violent recidivism consisted of any new criminal code conviction for an offense against the person with the potential for physical or psychological harm (e.g., assault, homicide, robbery), including sexual offenses. General recidivism consisted of a new conviction for any category of offense, be it violent or nonviolent. Offenses were coded in binary (yes 1, no 0) fashion. Conviction dates for first new offenses under a given category were also coded to track time to conviction to generate fixed follow-ups for data analysis (see Planned Analyses).

### Procedure

All files of the 574 persons declared NCRMD in the history of the province were examined for inclusion and exclusion based on either the availability of file information or recidivism data. Missing information was not assumed to be missing for any systematic reason—data were either missing due to age of file (i.e., older files contained less information, ink on onionskin paper was illegible) or the research assistant’s discretion (i.e., the reports on file did not comment on needed information) in consultation with the project principal investigator (PI)—and was excluded from analysis. Demographic, clinical, criminological, and VRAG-R risk variables were coded by the first author and a team of undergraduate research assistants from files located at the hospital, a community mental health outpatient facility, and the Alberta Review Board. In the minority of instances when the VRAG-R had already been coded by hospital psychology or psychiatry staff, the item and total scores were extracted for inclusion in this study in lieu of rerating the measure from file. The Alberta NCR project principal investigator, a registered psychologist with over 20 years experience working with forensic and correctional populations, provided training to the research assistants on study measures and oversaw all data collection. The first author also attended online VRAG-R training through the Global Institute of Forensic Research. The research assistants completed regular scoring validity checks and also had access to the principal investigator and senior researchers for questions.

To examine interrater reliability, 30 files (6.3% of the sample) were randomly selected and independently double coded. Per Cicchetti and Sparrow (1981), “excellent” interrater reliability for VRAG-R total scores was obtained via intraclass correlation coefficient, single measure, absolute agreement, one-way random effects model: ICC_A,1_ = .983, 95% confidence interval (CI) (.965, .992), *p* < .001; essentially the same results using consistency agreement were obtained, ICC_C,1_ = .984, 95%CI (.966, .992), *p* < .001. We also examined the interrater reliability of VRAG-R risk category ratings. Given that there are nine possible VRAG-R risk categories, agreement statistics should consider the degree of disparity between raters in assigning categorical risk ratings. For instance, consider two raters who rated Case A in bin 8 versus bin 9, respectively; this would be very different than had one rater assigned bin 1 and the other, bin 9. Weighted agreement statistics are intended for more than two ordered categories between raters to take such disparities into account that would otherwise be missed by simple binary “yes–no” agreement. Given that there were more than two ordered categories between raters for the VRAG-R bins we chose to compute weighted kappa (see Watson & Petrie, 2010), but also present the unweighted kappa (κ) for comparison purposes. We also computed Gwet’s AC1 and its weighted counterpart, AC2, using package “irrCAC (version 1.0)” (Gwet, 2019) through R 4.0. The following interrater reliability results for the VRAG-R bins were obtained: κ unweighted = .491, κ weighted = .865, Gwet’s AC1 = .516 (95%CI = .305–.728), percent agreement (binary) = .567, AC2 = .967 (95%CI = .946–.988), percent agreement (weighted) = .992. Of note, 56.7% (17/30) of ratings had the same VRAG-R risk bin, 40% (12/30) differed by 1 bin, and in one instance (3.3%, 1/30) did raters differ by two bins. In all, satisfactory interrater agreement was observed for VRAG-R continuous scores and the nine risk bins. VRAG-R scores in the total sample also demonstrated good internal consistency (Cronbach’s α = .84), despite the differential weighting and fairly heterogeneous content of the 12 items. Finally, omitted items were prorated following the protocol outlined by the instrument developers by creating a weighted average for missing values (up to four missing) that is added to the existing unprorated total score to create a prorated score.

### Planned Analyses

Data analysis proceeded over several steps. First, descriptive statistics and frequency distributions of VRAG-R scores and their respective bins were examined. This was completed for both scores via the entire population and separated by gender. Second, to ascertain the capacity of VRAG-R bin and total scores to discriminate recidivists from nonrecidivists, the discrimination properties of the VRAG-R were examined in the prediction of fixed 5-year, fixed 10-year, and overall (unfixed) violent and general recidivism via ROC analyses. ROC analyses generate an AUC statistic ranging from 0 to 1 representing the probability that a randomly selected recidivist will score higher on a given risk tool than a randomly selected nonrecidivist. With values of .50 representing chance levels of predictive accuracy, AUC values of .556, .639, and .714 represent small, medium, and large effect sizes, respectively, (Rice & Harris, 2005). These analyses were conducted in the population as a whole and the large male subsample; there were insufficient female recidivists to conduct predictive validity analyses with this subsample.

Third, to examine the rates of recidivism associated with VRAG-R scores and the generalizability of the VRAG-R norms to the current sample, calibration analyses were conducted. To do this, per Olver and Sewall (2018), logistic regression was used to model 5- and 10-year estimates of violent recidivism with specific VRAG-R scores. Logistic regression generates a constant (*B*_0_) which is the log odds of the recidivism base rate, and regression coefficients (*B*_1_), each representing the percent increase in the odds of a given outcome between adjacent scores on the measure. Specific scores on the tool under examination, in this case the VRAG-R, in conjunction with base rate information from the sample, can be used to estimate a specific score through the use of a logistic function: e^B0+B1xScore^/(1 + e^B0+B1xScore^) (Tabachnick & Fidell, 2007). These results were then graphically juxtaposed with the reported actual rates of violent recidivism observed for each of the nine risk bins of the VRAG-R.

A further critical step toward examining calibration was through computation of the *E*/*O* index (i.e., expected/observed index) per Hanson (2017; see also Gregório Hertz et al., 2021; Olver & Sewall, 2018). An *E*/*O* index results in a ratio of expected number of recidivists to the actual observed number of recidivists. With an *E*/*O* index of 1 representing perfect calibration, *E*/*O* index values below 1.0 indicate the underprediction of recidivism by the reference group, while values over 1.0 represent the over-prediction of recidivism (e.g., an *E*/*O* index value of 3 would indicate that the measure has overestimated three times the number of recidivists; an *E*/*O* index of 0.6 would indicate that the scale underpredicted 40% of observed recidivists). The *E*/*O* index is significant when the 95%CI does not overlap with 1.0 (Rockhill et al., 2003): $95\%\;\mathrm{CI}\;\mathrm{of}\;E/O\;\mathrm{Index}\;=\;(E/O)e^{\left(\pm 1.96\sqrt{1/O}\right)}$ . The *E*/*O* index was also calculated to compare the observed rates of 5-year recidivism for the Alberta NCRMD population to the expected rates of recidivism for the VRAG-R normative sample. Given that the Rice et al. (2013) sample is the normative sample upon which to interpret VRAG-R scores, we viewed it as quite critical to compare it to the Alberta NCRMD sample for the *E*/*O* index analyses, which would also be consistent with past practice (per Gregório Hertz et al., 2021; Olver & Sewall, 2018).

As a fourth and final point of investigation, the discrimination and calibration (excluding the *E*/*O* index due to sample and space considerations) analyses were repeated for the four most common diagnostic categories within the database (e.g., any psychotic disorder, any mood disorder, substance use disorder or SUD [present or ever] and antisocial personality disorder or ASPD, including traits).

## Results

### VRAG-R Descriptive Statistics and Frequencies

Table 1 provides the VRAG-R descriptive statistics for the Alberta-NCRMD population overall and by gender. The population average VRAG-R score was low (*M* = −6.8, *SD* = 19.0), particularly compared with the Harris et al. (1993) original VRAG construction sample (*M* = 0.91, *SD* = 12.9) and the Rice et al. (2013) VRAG-R normative sample (*M* = 3.6, *SD* = 12.5). Furthermore, males had significantly higher VRAG-R scores than females: *t*(136.934) = 6.138, *p* < .001, *d* = .69. VRAG-R bin number was significantly associated with gender (χ^2^ [8, *n* = 478] = 40.02, *p* < .001, φ = .29), with about the half of females scoring in bin 1, in contrast to about the half of males distributed among the first four bins.

**Table 1.** Descriptive Statistics and Bin Frequencies for VRAG-R Scores.

VRAG-R bin (range)	Overall		Female		Male	
	*N* (%)	*M* (*SD*)	*n* (%)	*M* (*SD*)	*n* (%)	*M* (*SD*)
1 (< −25)	109 (22.8)	−28.55 (3.3)	37 (46.3)	−28.86 (3.1)	72 (18.1)	−28.39 (3.4)
2 (−25 to −19)	76 (15.9)	−20.13 (2.1)	9 (11.3)	−20.22 (2.5)	67 (16.8)	−20.12 (2.1)
3 (−18 to −14)	55 (11.5)	−13.51 (1.7)	12 (15.0)	−13.67 (1.5)	43 (10.8)	−13.47 (1.8)
4 (−13 to −8)	56 (11.7)	−6.79 (2.2)	8 (10.0)	−6.25 (2.5)	48 (12.1)	−6.88 (2.2)
5 (−7 to 5)	43 (9.0)	−0.12 (2.0)	3 (3.8)	1.00 (2.0)	40 (10.1)	−0.20 (2.0)
6 (6 to 12)	46 (9.6)	7.43 (2.3)	7 (8.8)	7.71 (2.6)	39 (9.8)	7.38 (2.2)
7 (13 to 18)	25 (5.2)	14.32 (1.9)	1 (0.2)	12.00 (n/a)	24 (6.0)	14.42 (1.9)
8 (19 to 27)	34 (7.1)	21.47 (2.4)	3 (3.8)	22.00 (3.6)	31 (7.8)	21.42 (2.4)
9 (28+)	34 (7.1)	32.22 (3.8)	0 (0)	–	34 (8.5)	32.22 (3.8)
Total	478 (100)	−6.79 (19.0)	80 (100)	−16.61 (14.9)	398 (100)	−4.81 (19.1)
Min, Max	−34, 43		−34, 25		−34, 43	

*Note*. VRAG-R = Violence Risk Appraisal Guide–Revised; *SD* = standard deviation.

### Discrimination Properties of the VRAG-R for Violent and General Recidivism

Rates of recidivism for the Alberta NCRMD population were as follows: Violent recidivism 5.4% (22/405) 5-year, 7.7% (31/401) 10-year, 9.2% (44/476) overall; General recidivism 8.8% (36/405) 5-year, 13.2% (53/401) 10-year, 14.9% (71/476) overall. Owing to the small number of female recidivists (*n* = 1 recidivist) with a complete VRAG-R score, formal examination of the discrimination properties of the VRAG-R could only be completed for the male subgroup. As such, rates of recidivism for the male subgroup were as follows: violent recidivism 6.3% (22/346) 5-year, 9.1% (31/342) 10-year, 11.1% (43/389) overall; General recidivism 10.4% (36/346) 5-year, 15.4% (53/342) 10-year, and 18.0% (70/389) overall.

Table 2 provides AUC values for VRAG-R prediction of these recidivism outcomes in the overall Alberta NCRMD population and in the male subsample. VRAG-R total score and bin level significantly predicted general and violent recidivism in the aggregate sample and among males. In both groups, slightly higher predictive accuracy was observed for violent than general recidivism, as well as for total scores versus bin number. In the Alberta NCRMD population, AUC magnitudes were moderate to large for violence and moderate for general recidivism across VRAG-R measures. In the male subgroup, AUCs were slightly lower, evincing moderate predictive accuracy for all outcomes across follow-up, with the exception of small AUCs for general recidivism at the unfixed follow-up.

**Table 2.** Discrimination Properties of the VRAG-R for Violent and General Recidivism for 5-Year, 10-Year, and Overall Follow-Up: Aggregate Sample and Male Subsample.

VRAG-R measure and recidivism criterion	Aggregate		Males	
	AUC	95% CI	AUC	95% CI
Total score: violence				
5-year	.711***	[.604, .818]	.691**	[.579, .803]
10-year	.714***	[.624, .804]	.694***	[.600, .788]
Overall	.703***	[.626, .779]	.672***	[.590, .754]
Total score: general				
5-year	.682***	[.587, .777]	.662***	[.564, .760]
10-year	.674***	[.595, .754]	.653***	[.571, .736]
Overall	.654***	[.586, .722]	.621***	[.549, .693]
Bins: violence				
5-year	.691**	[.580, .801]	.670**	[.555, .784]
10-year	.698***	[.606, .709]	.677***	[.581, .773]
Overall	.691***	[.613, .770]	.659***	[.575, .742]
Bins: general				
5-year	.666***	[.571, .762]	.645**	[.547, .744]
10-year	.663***	[.584,.743]	.641***	[.559, .724]
Overall	.642***	[.573, .711]	.608**	[.536, .681]

*Note*. VRAG-R = Violence Risk Appraisal Guide–Revised; AUC = area under the curve; CI = confidence interval.**p < .01, *** *p* < .001.

### Calibration Properties of the VRAG-R for Violent Recidivism

**Figure 1.**
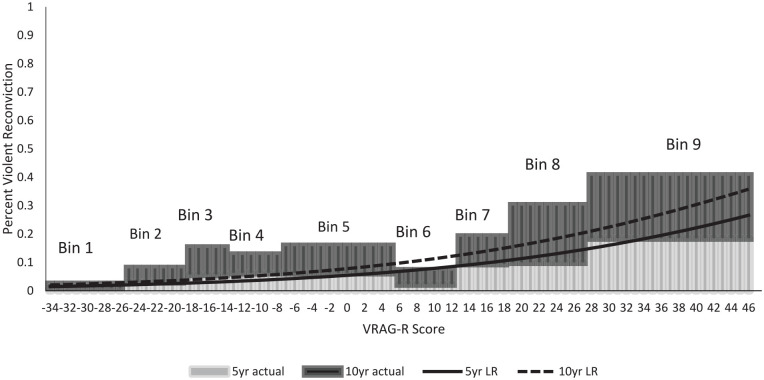
VRAG-R Calibration: Observed Rates of Violent Recidivism for the Nine-Bin Structure and Estimated Rates of Violent Recidivism Associated with Individual Scores over Fixed 5- and 10-Year Follow-Ups. VRAG-R = Violence Risk Appraisal Guide–Revised.

**Figure 2.**
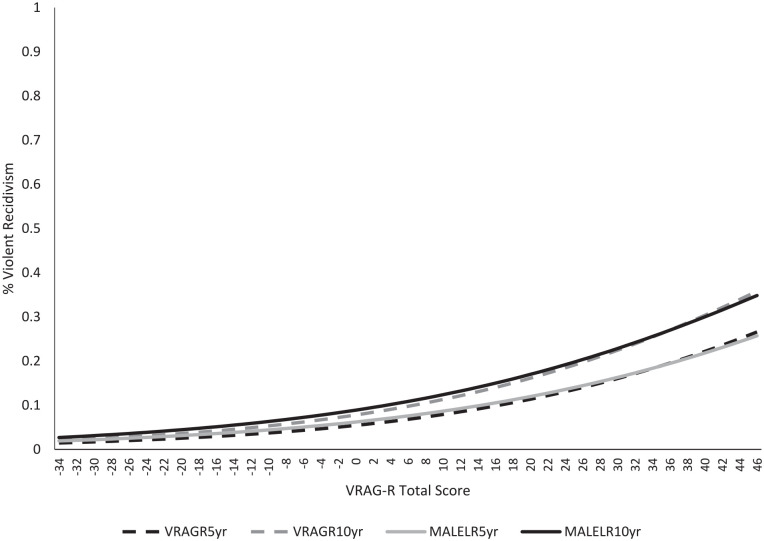
Logistic Regression Estimated 5- and 10-Year Rates of Violent Recidivism for all Possible VRAG-R Scores for the Overall Population (Dashed Lines) and Male Subsample (Solid Lines). *Note*. VRAG-R = Violence Risk Appraisal Guide–Revised; MALELR = male subgroup logistic regression curve.

The calibration properties of the VRAG-R for violent recidivism were examined through logistic regression and *E*/*O* index in the Alberta NCRMD population and large male subsample. First, logistic regression was used to estimate rates of 5- and 10-year violent recidivism associated with all possible VRAG-R scores. For each set of analyses, the Hosmer–Lemeshow goodness of fit tests were all nonsignificant suggesting acceptable calibration of the predictive models and that the logistic distributions provided a reasonable approximation of violent recidivism rates to warrant modeling. Results of the logistic regression generated the following terms for 5-year (*B*_0_ = −2.856, *B*_1_ = .040, *p* < .001) and 10-year (*B*_0_ = −2.471, *B*_1_ = .041, *p* < .001) violent recidivism. Figure 1 presents the trajectories of 5- and 10-year violent recidivism for all possible VRAG-R scores generated through use of the logistic function, juxtaposed with actual observed rates of violent recidivism for each bin. As seen in the figure, there are some fluctuations in observed rates due to small numbers of recidivists in some bins, underscoring the utility of logistic regression for estimating rates of recidivism associated with specific risk scores. When this exercise was repeated with the male subsample, generally identical trajectories of 5- and 10-year violent recidivism were observed (Figure 2, dashed lines) versus the Alberta NCRMD population as a whole (Figure 2, solid lines). The logistic regression terms for VRAG-R prediction of violent recidivism for the male subsample were as follows: 5-year (*B*_0_ = −2.717, *B*_1_ = .036, *p* = .002); 10-year (*B*_0_ = −2.328, *B*_1_ = .037, *p* < .001).

**Figure 3.**
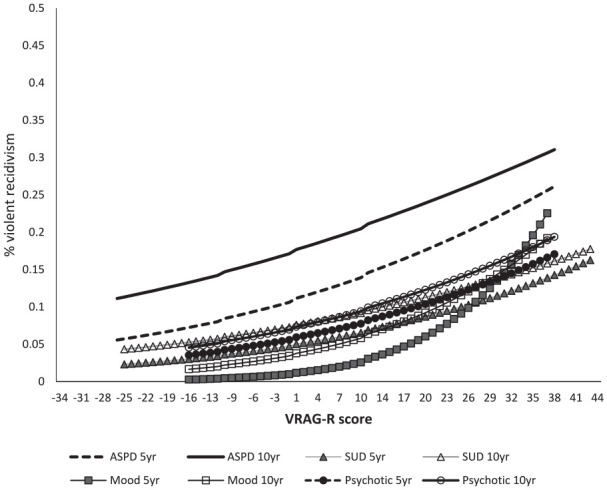
Logistic Regression Estimated Trajectories of 5- and 10-year Violent Recidivism for Observed VRAG-R Scores by Diagnostic Category. *Note*. VRAG-R = Violence Risk Appraisal Guide–Revised; SUD = substance use disorder; ASPD = antisocial personality disorder or traits.

*E*/*O* Indices were then computed as a formal examination of calibration for 5-year violent recidivism in the Alberta NCRMD population (Table 3, top half) and male subsample (Table 3, lower half), compared with the Rice et al. (2013) 5-year violence norms; 10-year data for the VRAG-R normative sample were not available at the time (Helmus, personal communication, March 13, 2019) and as such a 10-year *E*/*O* index could not be computed. VRAG-R scores substantially overestimated risk and corresponding rates of 5-year violent recidivism for the AB-NCRMD population, given that almost all E/O index values greatly exceeded 1 and were significant, and some values even reaching as high as 15 (Bin 6). Overall, the VRAG-R normative sample from Rice et al. (2013) overestimated 5-year violence risk by 4.6 times. In general, there was poor agreement between the expected and observed recidivism rates, with only two of the *E*/*O* index values being nonsignificant (bins 1 and 4), one of which was due only to the extremely small frequency of recidivists (bin 1, which was still overestimated by sevenfold). When the analyses were repeated for the male subsample, the same pattern of overprediction was slightly less extreme but still observed, with significant *E*/*O* index values observed for bins 6-9 and overall.

**Table 3.** E/O Index: Five-Year Rates of Violent Recidivism for Normative Sample (Rice et al., 2013) Compared with the Alberta NCRMD Population: Overall Sample and Male Only.

VRAG-R bin and range	5-year violent recidivism						
	*n*	Expected rates: VRAG-R norms		Observed rates: Current sample		*E*/*O* Index	W95% CI
		%	*n*	%	*n*		
Overall							
1 (≤ −25)	87	8.0	7.0	1.1	1	7.0	0.98, 49.7
2 (−24 to −19)	64	9.0	5.8	3.1	2	**2.9**	**1.89, 4.47**
3 (−18 to −14)	53	18.0	9.5	5.7	3	**3.2**	**1.06, 9.79**
4 (−13 to −8)	48	19.0	6.7	6.3	3	2.2	0.73, 6.73
5 (−7 to 5)	32	25.0	8.0	6.3	2	**4.0**	**2.60, 6.16**
6 (6 to 12)	43	37.0	15.9	2.3	1	**15.0**	**2.23, 112.89**
7 (13 to 18)	21	45.0	9.5	9.5	2	**4.7**	**3.06, 7.24**
8 (19 to 27)	30	58.0	17.4	10.0	3	**5.8**	**1.91, 17.75**
9 (28+)	27	80.0	21.6	18.5	5	**4.3**	**1.76,10.36**
Total	383	26.5	101.4	5.4	22	**4.6**	**3.04,6.95**
Male subsample							
1 (≤ −25)	62	8.0	5.0	1.6	1	5.0	0.70, 35.5
2 (−24 to −19)	58	9.0	5.2	3.4	2	2.6	0.65, 10.56
3 (−18 to −14)	42	18.0	7.6	7.1	3	2.5	0.83, 7.65
4 (−13 to −8)	42	19.0	5.9	7.1	3	2.0	0.66, 6.12
5 (−7 to 5)	30	25.0	7.5	6.7	2	3.8	0.95, 15.43
6 (6 to 12)	37	37.0	13.7	2.7	1	**13.7**	**1.92, 97.27**
7 (13 to 18)	20	45.0	9.0	10.0	2	**4.5**	**1.13, 18.27**
8 (19 to 27)	28	58.0	16.2	10.7	3	**5.4**	**1.78, 16.52**
9 (28+)	27	80.0	21.6	18.5	5	**4.3**	**1.76, 10.36**
Total	346	26.5	91.7	6.4	22	**4.2**	**2.77, 6.34**

*Note*. VRAG-R = Violence Risk Appraisal Guide–Revised; CI = confidence interval. Bolded *E*/*O* index and 95% CIs denote significance.

### Discrimination and Calibration Properties of the VRAG-R by Diagnostic Category

Discrimination and calibration analyses were repeated across broad diagnostic categories highly represented in this population: any psychotic disorder, any mood disorder, SUD (i.e., present or ever), and ASPD (including ASPD traits). Rates of recidivism by diagnostic subgroup were as follows: psychotic disorder, violent (5-year 5.4%, 18/334; 10-year 6.7%, 22/330; overall 8.3%, 32/387) and general (5-year 8.0%, 27/334; 10-year 11.0%, 36/330; overall 13.2%, 51/387) recidivism; mood disorder, violent (5-year 1.5%, 2/130; 10-year 3.1%, 4/129; overall 5.9%, 9/153) and general (5-year 4.6%, 6/130; 10-year 8.5%, 11/129; overall 12.4%, 19/153) recidivism; SUD, violent (5-year 5.6%, 12/216; 10-year 8.0%, 17/215; overall 8.8%, 22/251) and general (5-year 10.6%, 23/216; 10-year 15.3%, 33/215; overall 16.7%, 42/251) recidivism; and ASPD, violent (5-year 15.7%, 13/83; 10-year 21.7%, 18/83; overall 19.6%, 19/97) and general (5-year 18.1%, 15/83; 10-year 27.7%, 23/83; overall 26.8%, 26/97) recidivism.

Table 4 provides the results of ROC analyses to examine the discrimination properties of VRAG-R score and bin within each the four diagnostic subgroups. For persons with psychotic disorders (the largest diagnostic subgroup), VRAG scores and bins significantly predicted both outcomes irrespective of follow-up, with moderate effects for violence and small effects for general recidivism. For the mood disorders subgroup, predominantly large effects were observed for violence and moderate effects for general recidivism, however, the AUC magnitudes had some instability and wide CIs, particularly for 5-year outcomes due to the small number of recidivists. Finally, VRAG-R scores and bins had small and nonsignificant predictive effects for both sets of outcomes within the ASPD and SUD subgroups.

**Table 4.** Discrimination Properties of the VRAG-R for Violent and General Recidivism as a Function of Follow-Up and Diagnostic Category.

VRAG-R measure and recidivism criterion	Psychotic		Mood		SUD		ASPD	
	AUC	95% CI	AUC	95% CI	AUC	95% CI	AUC	95% CI
Total score violence								
5-year	.666*	[.546, .787]	.902	[.761, 1.00]	.646	[.468, .824]	.632	[.450, .814]
10-year	.658*	[.548, .768]	.771	[.576, .966]	.610	[.466, .754]	.587	[.423, .751]
Overall	.658**	[565, .751]	.710*	[.539, .881]	.604	[.482, .725]	.566	[.411, .711]
Bins violence								
5-year	.645*	[.520, .771]	.873	[.719, 1.00]	.625	[.449, .801]	.599	[.419, .788]
10-year	.637*	[.523, .752]	.766	[.605, .927]	.597	[.455, .738]	.555	[.394, .716]
Overall	.647**	[.551, .743]	.692	[.514, .870]	.597	[.477, .717]	.538	[.387, .689]
Total score general								
5-year	.636*	[.527, .745]	.886***	[.792, .979]	.608	[.473, .743]	.662	[.498, .825]
10-year	.633**	[.536, .731]	.697*	[.515, .880]	.586	[.476, .697]	.595	[.450, .741]
Overall	.609*	[.528, .690]	.642*	[.504, 780]	.570	[.475, .665]	.550	[.449, .685]
Bins general								
5-year	.620*	[.512, .729]	.870	[.767, .973]	.593	[.457, .728]	.635	[.471, .799]
10-year	.621*	[.526, .717]	.695*	[.518, .871]	.572	[.462, .682]	.560	[.416, .705]
Overall	.599*	[.518, .680]	.624	[.481, .767]	.558	[.463, .653]	.522	[.390, .654]

*Note.* VRAG-R = Violence Risk Appraisal Guide–Revised; SUD = substance use disorder; ASPD = antisocial personality disorder or traits; AUC = area under the curve; CI = confidence interval.

* *p* < .05, **p < .01, *** *p* <.001.

Finally, logistic regression was conducted to estimate the rates of 5- and 10-year violent recidivism associated with specific VRAG-R scores within each of the four diagnostic subgroups; the regression model statistics are presented in Table 5, the results of which paralleled AUC findings. Hosmer–Lemeshow goodness of fit tests was all nonsignificant for each diagnostic subgroup suggesting that the logistic distributions provided a reasonable approximation of violent recidivism rates to warrant modeling. Application of the logistic function using the VRAG-R predictor and constant values from Table 5 generated the trajectories of 5- and 10-year violent recidivism for each diagnostic subgroup (see Figure 3). Of note, only the VRAG-R scores populated within a diagnostic subgroup were used to generate the curves. The curve for 5-year violence within the mood disorders group is particularly steep, likely owing to the small number of recidivists. Otherwise, similar trajectories of 5- and 10-year violent recidivism were associated with VRAG-R scores for three out of the four diagnostic subgroups; the one exception was considerably higher rates of 5- and 10-year violence for the ASPD subgroup irrespective of VRAG-R score. That is, individuals with ASPD had higher rates of recidivism for a particular score than did members of other diagnostic subgroups, but still at rates substantially lower than the VRAG-R norms (Rice et al., 2013).

**Table 5:** VRAG-R Logistic Regression Prediction Models for 5- and 10-year Violent Recidivism by Diagnostic Category.

Regression model by outcome	*B*	*SE*	Wald	*p*	*e*^B^ [95% CI]	
Psychotic disorders						
5-year violence						
VRAG-R score	.032	.013	6.026	.014	1.032 [1.006, 1.059]	
Constant	−2.798	.247				
10-year violence						
VRAG-R score	.030	.012	6.471	.011	1.030 [1.007, 1.054]	
Constant	−2.566	.224				
Mood disorders						
5-year violence						
VRAG-R score	.089	.046	3.755	.053	1.093 [.999, 1.196]	
Constant	−4.528	1.126				
10-year violence						
VRAG-R score	.050	.026	3.658	.056	1.051 [.999, 1.106]	
Constant	−3.287	.537				
SUD						
5-year violence						
VRAG-R score	.031	.017	3.498	.061	1.032 [.998, 1.067]	
Constant	−2.972	.335				
10-year violence						
VRAG-R score	.023	.014	2.699	.100	1.023 [.996, 1.052]	
Constant	−2.523	.269				
ASPD						
5-year violence						
VRAG-R score	.028	.021	1.837	.175	1.028 [.988, 1.071]	
Constant	−2.103	.471				
10-year violence						
VRAG-R score	.020	.017	1.288	.256	1.020 [.986, 1.055]	
Constant	−1.558	.378				

*Note*. VRAG-R = Violence Risk Appraisal Guide–Revised; *SE* = standard error; CI = confidence interval; SUD = substance use disorder; ASPD = antisocial personality disorder or traits.

## Discussion

This study sought to examine the risk profiles and recidivism outcomes of individuals who had been found NCRMD in the province of Alberta. The study had two goals: first to investigate the population for potential gender differences, and second to examine the predictive accuracy (i.e., the discrimination and calibration properties) of the VRAG-R.

The Alberta NCRMD population was much lower risk than the construction or validation sample used to develop the VRAG (Harris et al., 1993) and VRAG-R (Rice et al., 2013*).* The current sample scored about a half standard deviation lower overall than the normative group, and the differences were even more marked for females. As expected, females, overall, did have a much lower total score and bin number frequency distribution than their male counterparts at approximately 2/3 *SD* difference. Indeed, nearly half of all the females’ total scores classified their risk within the first risk bin of the VRAG-R, while an approximate similar proportion of males was distributed among the first four risk bins. The findings indicated few criminological variables for females that would indicate a higher risk score (e.g., criminal history) in addition to more sociodemographic characteristics that may indicate stronger prosocial functioning. In all, it was apparent that females commit crime and violence at lower rates and have lower risk scores than their male counter parts, a result that was clearly demonstrated within this study

### Discrimination and Calibration Properties of the VRAG-R in an NCRMD Sample

Consistent with recent previous VRAG-R research (Gregório Hertz et al., 2021; Hogan & Olver, 2019; Olver & Sewall, 2018; Rice et al., 2013) strong recidivism discrimination properties were observed for the VRAG-R with respect to the overall sample and male subgroup. AUCs were slightly higher for the sample overall than in the male subgroup; this is due to the AUC being a rank ordered statistic, with females having both lower scores and lower rates of recidivism, which would result in a greater concentration of recidivists at the top end of VRAG-R scores and hence higher AUC values. Among diagnostic subgroups, VRAG-R bin and total scores had good predictive accuracy for persons with psychotic illness or mood disorder, however, accuracy was lower (small effects) for SUD and ASPD diagnoses. We believe this is attributable to SUD and ASPD diagnoses tending to be the exception rather than the rule in this forensic mental health sample (in contrast to correctional settings); not only were the diagnostic base rates lower, but ASPD and SUD are themselves inherently criminogenic and on their own accounted for higher rates of recidivism.

The results of calibration analyses demonstrated that rates of 5- and 10-year violent recidivism increased with VRAG-R scores. Formal comparison of observed violent recidivism rates within the current sample to those expected from the VRAG-R normative sample, however, demonstrated that VRAG-R scores substantially overpredicted future violence at all risk bands. The *E*/*O* index was significant in 7 out of 9 bin comparisons and violent recidivism was overpredicted in each bin by 2 to 15 times (or by 200% to 1500%)! In their federal correctional sample, Olver and Sewall (2018) found that *E*/*O* index comparisons on rates of 5-year violence with the Rice et al. (2013) normative sample were not significant on most comparisons, although all were still lower than 1.0, reflecting nonsignificant overprediction still (and by a smaller margin). The Olver and Sewall (2018) sample was higher risk on the VRAG-R than both the current sample and the normative sample, suggesting: (a) the VRAG-R to be better calibrated to a higher risk sample and (b) that there are unique risk-relevant features of the normative sample (e.g., unmeasured dynamic risk factors, criminogenically relevant mental health symptoms) contributing to higher recidivism estimates not captured by the VRAG-R. With regard to the latter point, the Rice et al. (2013) VRAG-R normative sample would appear to be high risk in other ways not measured by the VRAG-R and as such, possibly less representative of other correctional or forensic mental health populations in Canada; hence, generating poorer calibration with the Alberta NCRMD sample.

### Implications for Research and Practice

Taken together the results of this study have several clinical and correctional implications regarding policy, practice, treatment, and assessment within the Alberta NCRMD population. First, the study findings demonstrate strong discrimination properties for the VRAG-R, but significant issues with calibration in a parallel NCRMD sample. Although some caution should be exercised given that the Alberta NCRMD population was much lower risk, it is sobering that overprediction occurred even at the lowest risk VRAG-R bands and strongly indicates that baseline actuarial risk does not explain the whole picture. The VRAG-R normative sample also used a slightly more liberal recidivism criterion (i.e., violent charges/convictions or returning to hospital for a reason that would have otherwise resulted in a criminal charge for a violent offense as opposed to the higher threshold of conviction), which may partly account for some disparities. Recidivism base rates can also be impacted by other factors such as prosecutorial discretion in which charges to prosecute, law enforcement officer arrest decisions based on mental health presentation during police encounters, or access to legal representation (e.g., Legal Aid).

Second, future research should examine the predictive validity of the VRAG-R with female correctional or forensic mental health samples. Such research would not only aid knowledge about the predictive properties of the VRAG-R with women, but potentially increase the number of tools available for risk assessment and management applications with this population. Gender stratified norms, however, as seen with other tools such as the Level of Service/Case Managment Inventory (LS/CMI), would be essential to avoid perpetuating the problem of overpredicting violence risk.

Third, the present findings highlight: (a) the importance of local norms for risk assessment measures and (b) the importance of conducting risk assessments as an integrated, multimeasure, multisource process that does not depend solely upon one single measure to appraise risk or to make decisions. It is clear that the VRAG-R norms would generate considerably higher projections of rates of future violence attached to scores. As such, the present findings may be taken as a set of local Alberta NCRMD norms, which likely represent more realistic portrayals of risk. Even still, the VRAG-R should not be used as a standalone measure and is likely best complemented by a dynamic measure, and in practice this often occurs. For instance, Olver and Sewall (2018) found that a measure of sexual violence risk incremented VRAG-R predictions, and that logistic regression could be used to model recidivism estimates incorporating treatment change information. This study findings also support the assertion of Canadian researchers elsewhere (Goossens et al., 2019; Grantham, 2014; Charette et al., 2015; Haag et al., 2021; Lacroix et al., 2017) that there is very little evidence that supports the idea that legislation change was needed to protect public safety (i.e., Bill C-14), given the lower levels of risk than general correctional samples and low rates of recidivism.

### Strengths, Limitations, and Conclusions

This study has important strengths and limitations. One potential limitation is that this study was archival and retrospective in nature, and invariably, the researchers were at the mercy of quality and quantity of information available on file. In some cases, the files were simply limited and insufficient, particularly with older files (e.g., circa 1940–1970), and the VRAG-R could not be coded. Relatedly, not all files could be retrieved for individuals identified NCRMD and were similarly excluded from this study. Second, NCRMD cases resulting in an immediate absolute discharge were not captured by this database given that they would not have come under Alberta Review Board jurisdiction and no file would be created; importantly such cases are very rare, yet still, this small select portion of the NCRMD population was unaccounted for in this research. Finally, during the study’s 70-year catchment period many changes have occurred in NCRMD legislation, as well as diagnostic classification, such as the various iterations of the *DSM* since its inception in 1952; all of which raise a third possible limitation in the potential for legislative and diagnostic cohort effects.

A unique and important study strength is its representation of the entire population of persons found NCRMD in Alberta’s history who have come under the Alberta Review Board jurisdiction. Second, this study has some important firsts; it is the first cross-validation of the discrimination and calibration properties of the VRAG-R in an NCRMD population outside of Ontario. It is also the first examination of potential gender differences within this population within Alberta, specifically on an actuarial risk measure and recidivism outcomes. Finally, there are also core methodological strengths that support the integrity and stability of findings. Specifically, high-quality VRAG-R data with strong interrater agreement was collected, with comprehensive long-term outcome data captured; methodological and data conditions that are ideal to permit rigorous examination of the predictive properties of VRAG-R scores.

As understanding about individuals found NCRMD improves, policy and legislation improves, and assessment and intervention can be tailored to manage violence risk and improve client wellbeing. Given that review boards have been recently instructed to place public safety as their paramount consideration, the results of this study can help to refine how much risk should be allocated to those persons found NCRMD and create a better balance between civil liberties and public safety.
